# Interrogation of imaging-based interspecies dynamics in the oral microbiome

**DOI:** 10.1080/20002297.2026.2640344

**Published:** 2026-03-05

**Authors:** Zhenting Xiang, Zi Wang, Nikoo Ghasemi, Yan Wang, Jing Wen, Yuan Liu

**Affiliations:** a Laboratory for Oral Health Translational Research, Department of Oral Health Sciences, Maurice H. Kornberg School of Dentistry, Temple University, Philadelphia, PA, USA; b Department of Microbiology, Immunology, and Molecular Genetics, Geffen School of Medicine, University of California, Los Angeles (UCLA), UCLA AIDS Institute, Los Angeles, CA, USA; c Department of Orthodontics and Dentofacial Orthopedics, School of Dentistry, Zanjan University of Medical Sciences, Zanjan, Iran; d Section of Public and Population Health, School of Dentistry, University of California, Los Angeles (UCLA), CA, USA

**Keywords:** Spatial organization, oral microbiome, interspecies interaction, spatial omics, imaging, artificial intelligence, computational modeling

## Abstract

**Background:**

The oral cavity presents a highly dynamic environment where inter-microbial communications play a pivotal role. Understanding the spatial organization of microbial ecosystems has been highlighted on the microbiome and polymicrobial infection. Furthermore, cross-feeding and modulation by metabolites from the oral microbiota and host cells, such as lactate and reactive oxidative species, impact the stability and functionality of microbial communities. Traditional research focusing solely on the compositional aspects of these communities is insufficient to understand the sophisticated interactions.

**Methods:**

We evaluated recent advancements in imaging technologies, bolstered by multi-omics analyses and artificial intelligence (AI)-driven approachesinsights, to provide an more integrated understanding of the dynamics and function of the oral microbiome.

**Results:**

Real time imaging and resolution-enhancing methods at the single-cell level have unraveled the ecology and dynamics of microbial communities, indicating unique three-dimensional architectures and biogeographical patterns associated with disease status in polymicrobial interplays. Emerging computational techniques can account for the spatial features of oral microbiome by creating image-like representations that capture the complex relationships between host tissues and microbial communities. Spatial multi-omics, help address the limitations of single-cell sequencing, deciphering molecular mechanisms between species in these biogeographical patterns. To process the massive volume of imaging-based data, AI-assisted analysis enables complex dataset integration, predictive capacity, and personalized treatment, bringing a whole new level of understanding of the oral microbiome and its relationships with the host.

**Conclusion:**

In this review, we highlight recent imaging-based technologies used to study the spatial biogeography of interspecies and interkingdom relationships within oral microbial communities, focusing on how these interactions and functional/metabolic alterations associated with health and disease. We further outline limitations of AI-generated predictions and imaging-based observational data. Finally, we elaborate on potential biomarkers for early diagnosis and new effective therapeutic strategies to reshape microbial dynamics.

## Introduction

The oral environment is exceptionally complex, hosting nearly 1,000 species of bacteria, viruses, fungi, archaea, and protozoa [1]. Within this dynamic milieu, interspecies interactions influence both the structure and function of microbial communities. Networks of synergistic and antagonistic relationships within oral microbiome give rise to emergent properties such as persistence, stability, and large-scale spatial organisation that mediate the shift from a state of health to diseases [2].

Heterogeneous and non-random spatial organisation is repeatedly observed in natural microbial ecosystems, and the oral cavity is no exception [3]. Distinct microbial habitats with unique spatial patterns exist throughout the mouth, forming an ecological gradient from front to back, regardless of the underlying tissue type (teeth, alveolar mucosa, keratinised gingiva, or buccal mucosa) [4]. Microorganisms live side by side, stack on top of each other, and intertwined to form a complex interacting community [5]. Meanwhile, dense and highly structured microbial consortia on the human tongue exhibit taxon clustering in domains suggestive of clonal expansion [6].

Emerging research increasingly highlights the crucial role of spatial structures and interspecies connections, both physical and chemical, in shaping microbial ecosystems [7]. Physical proximity is vital for microbial interactions; direct cell-to-cell contacts govern large-scale biofilm architecture and create the spatial context for chemical signalling and metabolic cooperation [8]. These spatially organised interactions drive synergistic relationships that enhance colonisation, persistence, and pathogenicity. Specific physical associations among diverse species may facilitate direct interspecies nutrient exchange, while localised, metabolite-mediated chemical signalling enables non-contact communication and coordinated functional behaviours over longer distances [9]. Notably, alterations in these spatial organisations have been linked to oral diseases, with distinct three-dimensional architectures and biogeographical patterns observed under dysbiotic conditions. The shift from health to disease is further reinforced by a feedforward loop involving microbiota, inflammation, and host factors [9].

With advances in sequencing technology, the site-specific hypothesis has gained strong support. For example, a recent study analysing short-read metagenomic data for the abundant oral genus *Streptococcus* found that even closely related species predominantly inhabit different regions of the mouth [10]. However, most current sequencing-based approaches, while providing robust compositional data, inherently disrupt spatial context and fail to capture the interactive dynamics among microbes and between microbes and the host [11].

**Figure 1.**
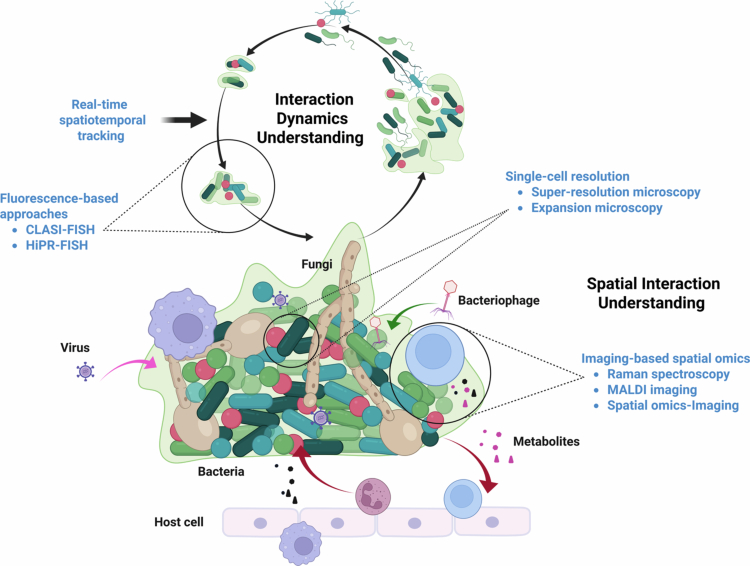
The need of imaging-based approaches in studying dynamic interspecies and microbe-host interactions within oral microbial communities. Created with BioRender.com.

Imaging techniques capable of resolving microbial dynamics across experimental scales and environmental conditions remain indispensable for dissecting spatial ecology in complex ecosystems [12]. Electron microscopy requires thin sectioning and lacks inherent taxonomic labelling, limiting its utility in community-scale spatial ecology studies. In contrast, optical microscopy, when combined with labelling techniques, offers unparalleled spatial resolution, real-time visualisation, and the ability to capture molecular and cellular interactions within their native microenvironments, making them indispensable tools for studying spatial dynamics (Figure 1). In this review, we synthesise advances that bridge spatial organisation and metabolic interactions in the oral microbiome. We highlight imaging-based technologies, complemented by multi-omics integration and AI-driven analytics, to advance the understanding of spatial organisation and metabolic interactions within the oral microbiome.

1. **Novel fluorescence imaging techniques reveal highly structured oral microbiota**

**Figure 2.**
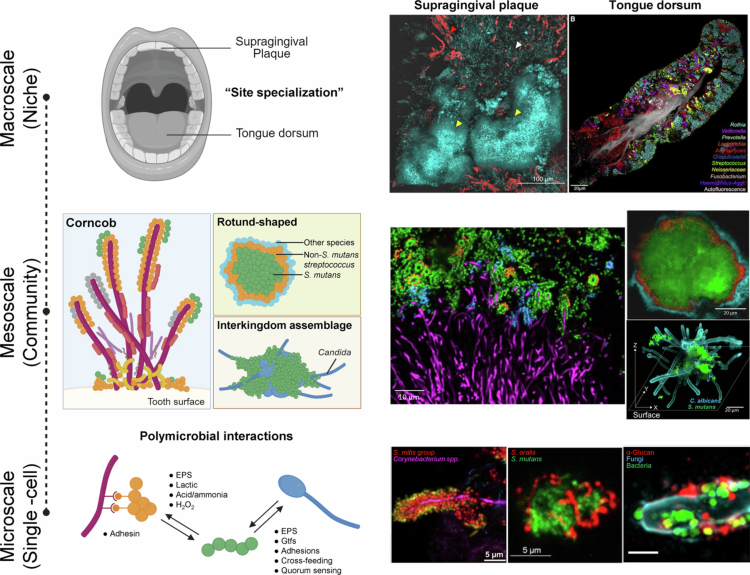
Biogeography of the oral microbiome at different scales. The right panel of the figure is adapted with permission from ref. [6,15–19]. Created with BioRender.com.

Building on the site-specific colonisation, imaging techniques have dramatically enhanced our ability to understand dynamic microbial organisation and quantify interactions *in situ*, providing critical spatial and temporal context that is often missing in bulk analyses [13]. Recent breakthroughs in fluorescence microscopy have been particularly transformative for the spatial mapping (biogeography) of oral microbiota. However, despite significant progress, fluorescence-based approaches have historically been limited by the number of taxa detectable in a single experiment [14]. Recent innovations incorporate highly multiplexed strategies, enabling the parallel interrogation of dozens to hundreds of microbial taxa or functional markers within a single sample (Figure 2).

When coupled with advanced optical systems, such as super-resolution microscopy or confocal laser scanning, fluorescence imaging reveals how different microbial taxa arrange themselves relative to one another and to host surfaces [20]. The microbiotas of dental plaque, the tongue dorsum, and the keratinised gingiva are the most distinctive from one another [21] (Figure 2 upper panel). Species-level imaging using CLASI-FISH (combinatorial labelling and spectral imaging–fluorescence in situ hybridisation) has revealed the dominance of certain species on the tongue dorsum, supporting sequencing-based evidence that most oral microbes are site specialists [6]. CLASI-FISH also facilitated the study examining plaque biofilm and suggests that *Corynebacterium matruchotii* may play an important role as a physical bridge between the base of the biofilm and its outer layers [17]. Interestingly, multiplex immunohistochemistry showed that fungi and bacteria mostly occupy separate dentinal tubules within carious dentin and rarely colocalize, which may partly reflect their limited likelihood of direct physical interaction [22].

Beyond site-specific localisation, mesoscale analysis further reveals that microbial architecture is governed by extracellular cues and biophysical properties, including chemical gradients (nutrients, oxygen, and antimicrobials), which both determine and are shaped by intercellular interactions and the interplay between cells and their environment. Such methods highlight microbial ‘hot spots’, where local interactions (e.g. metabolite exchange or competitive inhibition) may play a crucial role in shaping community assembly and function [6]. Across longitudinal samples, researchers observed recurrent, distinctive microarchitectures, such as clusters of *Lautropia* cells, indicating that oral microbiome spatial structure can be stable over time [23]. In dental plaque, *Corynebacterium* cells create habitat for other bacteria like a tree, some binding to the tips of filaments, for example, *Streptococcus* spp., where they may have direct access to the oral environment, such as saliva, forming a characteristic ‘corncob’ structure; others might be embedded deeper in the mass of biofilm, where they find a more sheltered, low oxygen environment that they need [17] (Figure 2 middle panel). This spatial organisation allows each microbial participant to interact with multiple, though limited, potential partners and is thought to promote the long-term stability of the community [18]. Another study of multispecies biofilms showed that *Streptococcus mutans* densely clustered in a ‘rotund’ extracellular scaffold, creating a localised low-pH zones that lead to enamel demineralisation which possible links spatial structure to the onset of caries [15] (Figure 2 middle panel). High phylogenetic resolution fluorescence *in situ* hybridisation (HiPR-FISH) further uncovered colocalization of *Rothia* and *Phocaeicola* cells in oral plaque samples, suggesting a potential metabolic synergy [24]. Spatial analysis in dental implant biofilms similarly revealed disease-associated architectures characterised by ecosystem instability: bacterial blooms form large patches, creating open niches that allow colonisation by new or opportunistic existing community members due to the absence of key functional roles. This supports a model of peri-implant dysbiosis that hypothesises altered biofilm spatial architecture facilitates the pathogenic colonisation [25]. By integrating confocal live-cell imaging and computational image analysis, Cho et al. [26] demonstrated that *S. sputigena* cells co-localised within *S. mutans*-derived EPS *α*-glucans, indicating *S. mutans* may facilitate the motile bacterium *Selenomona* colonisation and biofilm formation. Moreover, using dynamic tracking via flow-cell microfluidic to capture real-time imaging, researchers observed native cross-kingdom assemblages of *Candida albicans* and *S. mutans* with highly structured arrangement in saliva of children with severe tooth decay, exhibiting coordinated ‘leaping-like’ and ‘walking-like’ motions while continuously growing, a unique form of migratory spatial mobility that facilitates rapid biofilm expansion and potentially exacerbates tooth decay [19] (Figure 2 middle panel). Quantitative mesoscale image analysis can be extended to evaluate the immunogenic potential of discrete microbial consortia within the periodontal pocket. Such integrative approaches enable spatial mapping of dysbiotic clusters relative to tissue interfaces and local inflammatory microenvironments [27]. By linking biofilm architecture with host immune gradients and functional readouts, mesoscale imaging frameworks may therefore provide prognostic insight into disease progression and therapeutic responsiveness while refining mechanistic models of host-mediated microbial selection in periodontitis [28].

At an even finer scale, recent advances have enabled imaging at single-cell resolution (Figure 2 lower panel). Super-resolution confocal imaging with Airyscan detected *α*-glucans on bacterial and fungal surfaces within interkingdom assemblages in saliva from caries-active patients, highlighting a complex bacteria-fungi-EPS biostructure associated with early childhood caries [19]. However, in most cases, densely packed biofilms hinder the analysis of individual cells and their interactions. Heterotropic expansion microscopy addresses this challenge by coupling the microbial community to a swellable polyelectrolyte gel while leaving the cells themselves unswollen. Isotropic gel expansion decrowds the cells, enhancing spatial resolution with conventional microscopes and providing an unprecedented view of microbial heterogeneity [29]. This improved spatial detail has revealed distinct species-level interactions; for example, *Fusobacterium nucleatum* shows stronger adhesion to *Streptococcus sanguinis* than to *S. mutans*, suggesting specific preferences that may influence community assembly. Although significant progress has been made, microbiome imaging remains fundamentally constrained by complex microbial communities and the limited number of spectrally distinguishable fluorophores. Sequential FISH methods can markedly expand multiplexing, and error-correction strategies may further improve target-identification accuracy, enabling finer-resolution spatial mapping of oral microbial communities [30]. In addition, there is lack of research leveraging these highly multiplexed spatial imaging platforms to elucidate how microbial dysbiosis in one oral niche affects ecological balance in other niches. Using periodontal dysbiosis and tongue as an example, by simultaneously resolving taxonomic identity, spatial proximity, and functional markers across multiple oral habitats, these methods can reveal whether dysbiotic consortia or periodontal pathogens directly colonise on the tongue or reshape its microbial ecology through metabolite diffusion, inflammatory signalling, or host-derived factors. In turn, spatial analysis can further identify niche-specific microbial reservoirs on the tongue that may sustain periodontal pathogens or facilitate recolonisation of the subgingival environment. Importantly, mapping microbial co-colonisation and metabolic gradients across niches enables the identification of transmission pathways and inter-niche microbial networks that are obscure in the bulk sequencing approaches. Such insights are critical for redefining diseases not solely as a site-confined disease but as a spatial interconnected oral ecosystem disorder, thereby informing targeted interventions that disrupt pathogenic cross-niche interactions.

Admittedly, beyond physical interactions, the biochemical mechanisms underlying microbial spatial organisations require further validation. Key questions remain as to how metabolic exchanges are coordinated in the presence of oral pathogens or commensals, and whether polymicrobial interactions influence the growth dynamics of the spatial structure. Future studies using *ex vivo* and *in vivo* polymicrobial models are needed to investigate spatiotemporal dynamics within complex communities (Table 1). In addition to traditional *in vitro* studies, *in situ* approaches such as imaging-based spatial omics offer powerful tools to uncover these mechanisms.

**Table 1.** Summary of the insights and potential future directions for imaging-based approaches in oral microbiome research.

Imaging methods	Observations	Speculations for the interpretation	Limitations	Further validation suggestions
CLASI-FISH	*Corynebacterium matruchotii* grows in outward filaments that form hedgehog-like structures. [17]	Specific bacterial taxa act as physical bridges for biofilm architecture.	Inability to differentiate specific interaction vs. nonspecific adhesion.	Use realistic *in vitro*/synthetic-community models to perform biochemical and metabolic assays to assess interactions among taxa; Apply genetic or perturbation experiments to validate interaction; Conduct comprehensive analysis of spatial, temporal, and inter-individual variation.
Multiplex immune fluorescence staining/fluorescence microscopy	In caries dentin, Gram-positive bacteria and fungi colonise separately. [22]	Spatial separation of bacteria and fungi reflects the low probability of direct physical interaction.	Separation driven by tubule-size constraints from true interaction patterns; Unresolved cross-kingdom synergy or antagonism across caries stages.	Perform temporal or longitudinal co-culture assays to probe interspecies synergy or antagonism across disease stages; Apply perturbation or genetic assays to dissect microbe-microenvironment feedback; Quantify biochemical and nutrient gradients across spatial domains.
	On the tongue dorsum, individual taxa occupied distinct spatial domains, forming characteristic single-taxon patches within the community. [6]	Local chemical gradients (e.g. nutrients, oxygen, antimicrobials) likely guide microbial assembly and activity, shaping community dynamics.	Complex selective growth influenced by microenvironmental heterogeneity; Unresolved feedback between spatial stratification and local biochemical gradients.	
Non-invasive 3D *in situ* pH measurement/multiphoton confocal microscopy	The rotund-shaped community formed highly acidic microenvironments that promoted enamel demineralisation. [15]	Rotund architecture may create virulent hotspots at the biofilm–tooth interface.	Unverified impact of rotund architecture on *in vivo* virulence; Unproven relevance of this framework to severe childhood caries.	Validate rotund architecture in rodent caries models; Assess *in vivo* virulence linked to architectural features.
HiPR-FISH	Colocalization of *Rothia* and *Phocaeicola* in dental plaque [24].	*Rothia–Phocaeicola* colocalization may indicate metabolic synergy between these two genera.	Physical proximity without proof of metabolic exchange and function validation; No direct evidence of metabolite transfer or mutual benefit.	Track metabolite exchange with stable isotope probing; Confirm mutualistic or metabolite dependencies with functional assays. Conduct metabolic profiling or transcriptomic analysis; Use perturbation experiments to test causal relationships between structure and function.
	Peri-implantitis biofilms tend to form large clusters dominated by a single bacterial genus with reduced structural organisation [25].	Altered biofilm architecture creates niches that promote pathogen colonisation and destabilise the microbial community.	Spatial patterns without functional mechanistic validation; Unsubstantiated causal links between spatial organisation and community/host behaviour.	
Flow-cell microfluidic/Super-resolution real-time confocal imaging	Entrapment of *Selenomonas sputigena* in streptococcal exoglucans abolishes its motility but promotes active proliferation. [26]	*S. sputigena* engages in a cooperative interaction with *S. mutans* that exacerbates biofilm virulence.	Spatial patterns with limited mechanistic validation.	Future studies are needed to investigate the mechanisms underlying these interactions (e.g. Gtf with *S. sputigena*, glucan-fucose interactions, etc).
	Structured interkingdom assemblages display coordinated ‘leaping’ and ‘walking’ motions while continuously growing. [19]	Mobile multicellular interkingdom communities promote microbial spatial spreading across surfaces.	Lack of mechanistic experiments for mobile motion at molecule level.	Conduct *ex vivo* and *in vivo* polymicrobial models to confirm spatiotemporal dynamics. Apply genetic tools to understand microbial expanding mechanism.
Heterotropic expansion microscopy	*Fusobacterium nucleatum* shows stronger adhesion to *Streptococcus sanguinis* than to *S. mutans.* [29]	The close association between *F. nucleatum* and *S. sanguinis* is key interspecies interaction shaping supra- and subgingival plaque.	The underdetermined specific binding mechanisms.	Identify receptor-ligand binding pairs; Test adhesion mechanisms using gene-editing tools; Perform adhesion-blocking assays to confirm specificity.
Raman	Non-invasive visualisation enables species-specific biochemical features cross different biofilm regions. [31] [32] [33]	Proteins, lipids, and polysaccharides may serve as biochemical markers of bacterial proliferation and biofilm maturation.	Limited molecular specificity of spectral signatures; Biochemical species inferred, not directly measured.	Use functional assays of viability, metabolite activity, and adhesion to verify spectral features; Combine spectroscopy with multi-omics and high-resolution imaging to confirm that biochemical spectral changes match structural changes; Directly measure key species using chemical probes. Use oral relevant microbial culture systems.
	Dynamic changes of bacterial morphology can be mapped with real-time monitoring of nucleic acids, proteins, and lipid degradation. [34] [35]	Drug treatment or surface chemistry generates chemicals that disrupt metabolism and damage essential biomolecules, leading to bacteria lysis.	Limited mechanistic specificity of spectral signatures; Reactive species inferred, not directly quantified. No specific application in oral cavity.	
	Ultra-sensitive detection of dynamic biochemical changes reveal dose-dependent biofilm disruption and host metabolic shifts during viral replication [36].	Distinct Raman features can serve as biomarkers of virus-induced biofilm disruption and host metabolic reprogramming.	Highly overlapped spectral features with limited molecular specificity; Biochemical changes inferred, not independently validated; No specific application in oral cavity.	
MALDI-IMS	Spatially distinct molecular signatures across biological systems reveal biomolecular heterogeneity that links to cellular behaviour. [37] [38]	Spatially resolved molecular features can serve as functional signatures of biological states and help predict disease progression, cellular interactions, or biofilm stages.	Limited molecular specificity due to overlapping signals; Lacks orthogonal confirmation of MALDI-derived markers; Spatial patterns do not establish functional causality.	Include orthogonal MS or immuno-based confirmation of MALDI-identified molecules; Test causal relationships with perturbation studies (gene knockdown, inhibitor treatments, microbial QS disruption).
	Concentrated, interface-localised quorum-sensing metabolites are detected at the boundary between *S. aureus* and *P. aeruginosa* [39].	IR-MALDI increases ionisation of low-abundance biomolecules, improving bacterial molecular mapping.		
metaFISH	Symbiont-specific metabolite micro-domains map precisely to each symbiont’s gill tissue niche. [40]	Distinct spatial metabolite distributions represent symbiont-specific functions and compartmentalised host-microbe interactions.	Putative metabolite identification due to overlapping m/z features and limited structural resolution; Correlative not precise spatial co-localisation; No specific application in oral cavity.	Combine with species-resolved assignment with symbiont-targeted isotope labelling; Distinguish more metabolites with structural confirmation using LC–MS/MS, MS^n^ fragmentation, or NMR; Evaluate function with perturbation assays.

2. **Imaging-based spatial omics decodes the spatial organisation and interspecies interactions**

Metabolites act as nutritional currencies and communication signals within oral biofilms, enabling cross-feeding and collective resilience [41]. They also modulate host responses by triggering inflammation through lipopolysaccharides and peptidoglycan or promoting immune tolerance via tryptophan derivatives and short-chain fatty acids from *Porphyromonas* and *Fusobacterium*, which are commonly found in periodontal pockets [42]. However, traditional approaches for studying metabolites in complex communities have relied largely on bulk extraction and analysis, which destroy the sample and eliminate spatial context. These methods average signals across diverse cell populations and microenvironments, obscuring critical metabolic heterogeneity and interactions that are spatially organised within biofilms. In contrast, emerging imaging-based spatial omics tools allow direct visualisation and quantification of metabolites distributions within intact microbial communities.

To understand how metabolites mediate interspecies interactions within the oral cavity, it is critical to resolve their spatial distribution and cell-specific activity within intact communities. Raman Spectroscopy (RS), a vibrational spectroscopic technique that leverages the Raman effect, can detect and map metabolites at single-cell resolution. Raman spectra obtained from biofilms of different cariogenic *streptococci* revealed that each species produced a distinct spectral profile, especially in regions corresponding to lipids, amide proteins, and carbohydrates [31]. Notably, recent advances like stimulated Raman scattering (SRS) microscopy have achieved sub-micrometre resolution and video-rate imaging speeds in live cells and tissues [32]. *Pseudomonas aeruginosa* biofilms, where stratified metabolic activity across biofilm depth was visualised with high spatial resolution using SRS imaging of carbon-deuterium bonds, revealed phenazine-dependent biosynthetic hotspots in hypoxic subregions [33]. Furthermore, Raman spectral markers can serve as indicators of biofilm response to treatments and detect specific metabolite-driven interactions, as illustrated by the reduced Raman peak observed in *S. mutans*-rich dental biofilms treated with quaternary ammonium silane, enabling real-time monitoring of biofilm breakdown during antimicrobial treatment [35]. Using *in situ* Raman microprobe, peroxynitrite was observed to be produced by the *Porphyromonas gingivalis* on an antibacterial bioceramic surface, providing insight into location-specific oxidative stress chemistry within the biofilm [34]. Such observations highlight localised chemical activity but do not define causality in the absence of genetic or biochemical manipulation.

Surface-enhanced Raman scattering or spectroscopy (SERS) is a powerful technique combining nanotechnology and biomedicine, using plasmonic metallic nanostructures to detect molecular fingerprints with ultra-high sensitivity-even at the single-molecule level. Nanolaminated plasmonic crystals (NLPCs) with dense Au-SiO₂-Au nanogap hotspots enabled highly sensitive, real-time monitoring of living *Pseudomonas syringae* biofilms under phage infection. The platform captured dynamic biochemical changes, revealing virus dose-dependent biofilm disruption, cell lysis, and host metabolic shift during viral replication [36]. Similarly, SERS-based nanoplasmonic platforms could be applied to monitor the spatiotemporal dynamics of oral *Streptococcus-Veillonella* interactions, enabling real-time visualisation of metabolic exchanges (e.g. lactate consumption, short-chain fatty acid production) and biofilm development under therapeutic or ecological perturbations.

Interspecies and intraspecies interactions in the oral environment often involve diverse, untargeted metabolites or signalling molecules, requiring broad, discovery-driven mapping of the metabolic landscape that exceeds the molecular coverage achievable by RS. Matrix-assisted laser desorption/ionisation imaging mass spectrometry (MALDI-IMS) meets this need by simultaneously detecting and mapping hundreds to thousands of molecules in a single scan, directly from biological surfaces without labelling or targeting. By raster-scanning across a sample, MALDI-IMS generates a mass spectrum at each pixel, which can be computationally reconstructed into ion images showing the intensity of specific peaks across the sample surface. Unlike traditional assays that lose spatial context, MALDI-IMS preserves the molecule layout intact, providing label-free, spatially resolved metabolomic profiles. In oral cancer, MALDI-IMS identified localised biomarkers such as LRP 6 in oral squamous cell carcinoma [37], demonstrating its spatial power but not functional causality.

Building on the experience in cancer diagnosis, MALDI-IMS has also been explored to understand spatial chemistry in biofilms. Researchers monitored *N*-acyl-homoserine lactones (AHLs) production during *Pseudomonas putida* biofilm development, demonstrating uniform AHL signals distribution at early stage. As the biofilm matured, AHLs shifted to the peripheral accumulation of metabolites such as quinolones and pyochelin, correlating with swarming motility at the expanding edges [38]. Moreover, advanced instrumentation such as high-resolution MALDI or post-ionisation techniques enabled mapping of interspecies metabolites exchanged between competing adjacent bacterial colonies. A pulse infra-red (IR)-MALDI-IMS revealed that unique quorum-sensing molecules were strongly upregulated and concentrated at the immediate contact zone in co-cultures of *Staphylococcus aureus* and *P. aeruginosa*, where molecules produced by one colony diffused into the territory of the other [39]. These spatially resolved insights into microbial metabolism are directly relevant to oral biofilms, which also exhibit microscale zones of antagonism and cooperation, such as acidogenic *Streptococcus* species adjacent to acid-sensitive commensals, or bacteria-fungal interfaces in candidal infections. Thus, MALDI-IMS could be applied to dental plaque or mucosal biofilm samples to discover metabolites that distinguish pathogenic microenvironments from those associated with health, providing hypotheses that require downstream functional testing.

Building on this capability, emerging multimodal platforms further enhance spatial resolution and molecular identification. Advanced technologies, such as a spatial metabolomics pipeline (metaFISH) [43], have been a pioneering application combined MALDI-IMS with fluorescent microscopy to link chemistry with microbial identity. By overlaying MALDI maps with FISH on the same tissue section, metaFISH enables simultaneous visualisation of spatial distribution of metabolites and microbial taxa at micrometre to single-cell resolution [44]. Using this approach, the spatial metabolome of a deep-sea mussel and its intracellular symbiotic bacteria were resolved at the scale of individual epithelial cells, demonstrating metabolite exchange patterns that define symbiotic micro-niches [40,44]. Applied to oral systems, metaFISH could map metabolite gradients and microbial localisation within dental plaque or mucosal biofilms. This capability is particularly relevant for dissecting species-specific interactions and bacterial coaggregation patterns by directly linking physical microbial associations with localised metabolic activities. Notably, coaggregation involving probiotic strains within oral biofilms has been recognised as an important factor of dysbiosis and biofilm immunogenicity [45], underscoring a critical need in our understanding of how spatial organisation and local metabolic microenvironments mediate probiotic function *in situ*. By simultaneously visualising bacteria strains, their coaggregating partners, and associated metabolite signatures, metaFISH could address this gap by elucidating how probiotic incorporation into oral biofilms modulates community metabolism, host-relevant metabolites, and inflammatory potential. More broadly, this approach could uncover species-specific metabolic interactions, identify metabolite-based biomarkers, and monitor the effects of antimicrobial or probiotic interventions on oral metabolic homoeostasis. Together, this integrative imaging framework holds strong potential for dissecting microbe-host and microbe-microbe interactions in oral biology.

**Figure 3.**
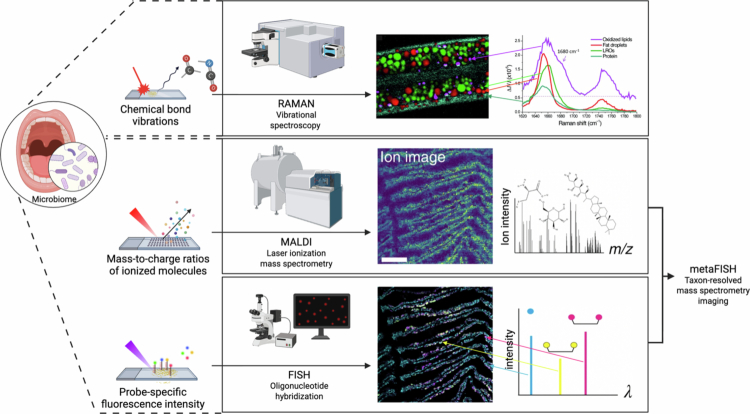
Overview of complementary spatial imaging techniques for mapping microbial organisation and metabolic features in the oral microbiome. Raman, MALDI mass spectrometry imaging, and FISH fluorescence-based approaches provide distinct but complementary spatial information on chemical composition, metabolite distributions, and microbial localisation. Scale bar, 150 µm. The imaging results are adapted with permission from ref. [40,46] Created with BioRender.com.

RS, MALDI-IMS, and metaFISH are complementary tools that allow unprecedented visualisation of metabolites *in situ* (Figure 3). Together, these spatial omics approaches enable researchers to visualise the chemical architecture of oral microbial communities; in fact, we can detect where acids are produced, showing where signalling molecules concentrate, and how all these chemical factors align with the microbial organisation; however, these data remain correlational and cannot reveal mechanistic interactions without complementary functional validation (Table 1). These findings will allow us to develop precision tools to modulate the microbiome and nudge the ecology of the mouth toward a healthy state.

3. **Artificial intelligence and computational techniques revolutionise the spatial structure of microbiome research**

**Figure 4.**
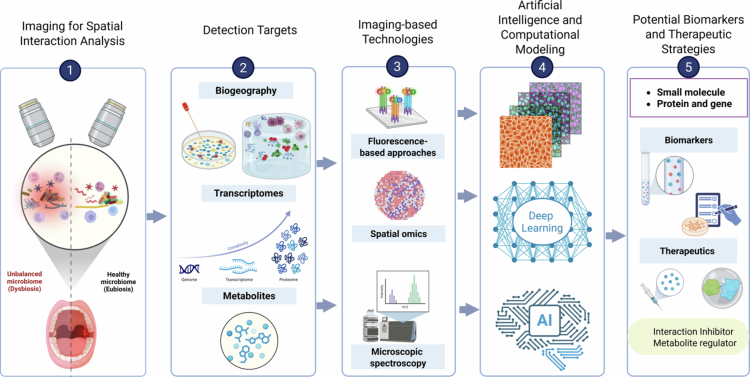
Advances in multimodal imaging, AI-driven analytics, and multi-omics integration in oral microbiome research. Created with BioRender.com.

Imaging-based assays now routinely yield terabytes of multi-channel, three-dimensional data, so extracting biological meaning increasingly depends on purpose-built analytic pipelines rather than manual inspection. Machine-learning frameworks are proving especially powerful. Figure 4 presents how AI and machine learning methodologies have been widely implemented in imaging data science and illustrates how the computational framework integrates into the overall analytical workflow across imaging platforms. Borowa et al. [47] explored bacterial species classification from polyculture microscopic images using multi-instance learning (a machine learning approach that handles grouped unlabelled data), bypassing the need for time-consuming monoculture preparation. This method enables faster and more interpretable diagnosis in complex microbial communities. Hahm et al. (2024) employed spatially resolved imaging (HiPR-FISH) to visualise the ecological structure of oral biofilms, demonstrating how pathogenic consortia in peri-implantitis form tightly clustered niches.

Imaging-based spatial omics is revolutionising microbiome research by providing direct insight into the interactions between microbial communities and their host environments. Spatial host–microbiome sequencing (SHM-seq) is an all-sequencing-based approach closely integrated with deep learning. It captures tissue, polyadenylated RNAs, and bacterial 16S rRNA directly from tissue by modifying a spatially barcoded glass surface, enabling simultaneous capture of both bacterial 16S rRNA and host transcripts [48]. Our ability to model these complex spatial structures has significantly improved with the emergence of artificial intelligence (AI)-assisted tools and machine learning techniques. For example, PM-CNN (Phylogenetic Multi-path Convolutional Neural Network) [49] models phylogenetic relationships using multi-layer hierarchical clustering of microbes and therefore provides more accurate microbiome-based disease prediction. Density-based unsupervised machine learning techniques can focus on spatial clustering. Different multi-omics approaches that integrate multiple data streams generate massive and complex data sources. The newly developed AI-powered, biology-inspired, multi-scale modelling framework may pave the way for a new level of understanding of spatial structure [50].

Moreover, the unique oral ecological environment, characterised by diverse niches such as saliva flow, mucosal surfaces, and tooth structures, gives rise to highly complex imaging datasets. These complexities underscore the need for advanced AI applications to integrate and interpret data from such heterogeneous sources. Pais et al. [51] developed predictive models using automated machine learning techniques to integrate datasets from saliva and subgingival biofilms. Their approach identified combinations of clustered microbial species to predict peri-implantitis. Similarly, Zhao et al. [52] used a deep learning model, PSPNet (a model for image segmentation), to capture complex microbial patterns beyond individual taxa. This method can process complex datasets, capture detailed context, and provide a comprehensive assessment of the oral microbiome for disease diagnosis.

Despite the promise of AI in oral microbiome research, the utility of these models depends heavily on data quality, diversity, and interpretability. Many studies report high predictive accuracy [51,53,54], yet sample size limitations, cohort diversity, and lack of experimental validation constrain their translational relevance. As noted in several prior studies [51,53,54], different metrics have been used to evaluate model performance. For example, Ding et al. [54] used Pearson’s correlation coefficient to assess the agreement between predicted and observed results. Zhao et al. [53] reported low mean absolute error to demonstrate prediction accuracy alongside computational efficiency. Importantly, Pais et al. [51] reported high sensitivity, reaching up to 95% in saliva samples, but relatively lower specificity in classification performance. To ensure that AI-generated predictions reflect true biological phenomena, complementary *in vitro* experiments and *in vivo* studies (e.g. animal models or clinical cohorts) are essential. Integration of host responses and spatial microbial organisation further underscores the need for experimental confirmation [24,55].

4. **Potential biomarkers for early diagnosis and therapeutic strategies to reshape microbial dynamics**

Therapeutic modulation of oral microbiome will depend on an accurate understanding of how microbial communities dynamically-spatially change over time and which interspecies interactions are required for maintaining healthy microbiome. To achieve this, incorporating biogeographical data will be key to informing and refining studies that examine the metabolic underpinnings of oral microbial ecology [13].

Several metabolites have been identified as potential biomarkers for dysbiosis and disease susceptibility. For instance, galactose utilisation, resulting from streptococcal sugar metabolism, may alter the physiology of *P. gingivalis*, impairing its ability to form biofilms and interact with host tissues [56]. Para-aminobenzoic acid (PABA), a metabolic product of *S. gordonii*, modulates *P. gingivalis* virulence by altering exopolysaccharide composition and fimbrial expression [57]. Butyrate, a short-chain fatty acid (SCFA) produced by *P. gingivalis*, *Tannerella forsythia*, and *F. nucleatum*, plays a significant role in immune suppression by reducing neutrophil phagocytosis and downregulating intercellular adhesion molecules (ICAM-1), thereby promoting chronic inflammation in periodontal disease [58]. The spatial distribution of these metabolites provides critical insights into disease progression and identified opportunities for targeted intervention.

Given the critical role of metabolites in microbial dynamics and host immune responses, targeting key metabolic pathways has emerged as a promising therapeutic strategy in oral health. Probiotic approaches, such as *Lactobacillus* and *Bifidobacteria* species, can modulate lactate metabolism to counteract acidification and reduce caries risk [59]. In periodontal disease, *Limosilactobacillus reuteri* has also been studied as an adjunctive therapy, with emerging evidence indicating that its probiotic benefits extend beyond direct antagonism of individual pathogens such as *Fusobacterium nucleatum*, instead reshaping biofilm composition and immunogenicity more broadly [45]. Beyond lactate metabolism, oral nitrate-reducing bacteria on the tongue dorsum and within subgingival plaque contribute to the nitrate-nitrite-nitric oxide pathway, with potential implications for nitric oxide bioavailability and local redox and inflammatory balance [60]. In this context, spatially resolved metabolomic imaging approaches such RS, MALDI-IMS, and metaFISH could directly evaluate the probiotic potential by linking niche-specific colonisation with localised metabolic gradients and host-microbe signalling *in situ*.

Together, these findings highlight a conceptual shift toward precision modulation of microbial metabolism as a means to restore oral homoeostasis. Nanomaterials, specifically those generating or scavenging reactive oxygen species (ROS), are being explored for their dual ability to disrupt pathogenic biofilms while mitigating oxidative stress. Protein therapies targeting specific metabolic enzymes, such as lactate oxidase or catalase [61], are also under investigation for their potential to restore redox balance and inhibit pathogen growth. In particular, enzyme-functionalized nanoparticles stand out as a highlight treatment [62], combining the precision of enzymatic activity with the stability and delivery advantages of nanotechnology. These systems can degrade harmful metabolites, such as butyrate or lactate, while simultaneously modulating microbial interactions and immune responses, offering a multifaceted approach to restoring oral microbial balance. This convergence of metabolic and nanotechnological strategies represents a transformative step toward precision-targeted therapies that not only address oral diseases but also have the potential to improve systemic health outcomes.

Importantly, the efficacy of these metabolism-targeted strategies is intrinsically linked to the spatial organisation of microbial communities. Metabolite gradients, microenvironments, and host-microbe signalling are not uniformly distributed but instead localised microbial interactions within spatial structures. As such, therapeutic outcomes cannot be fully captured by compositional analysis alone, underscoring the need for evaluation frameworks that reflect both function and spatial context. While current oral microbial dysbiosis indices, such as periodontal indice, primarily rely on compositional shifts in relative abundance, emerging spatial imaging techniques offer an opportunity to refine these metrics by incorporating architectural and proximity-based parameters [63]. High-plex fluorescence imaging and analysis can resolve contact-dependent interactions between homoeostatic and dysbiotic taxa, biofilm depth gradients, and epithelial interface engagement that are not captured by sequencing alone. Such spatially informed descriptors integrating interspecies proximity, vertical stratification, and tissue invasion, may enhance the diagnostic and prognostic sensitivity of existing microbial indices [64]. A similar framework may be extended to oncologic settings, where integrative spatial omics analyses of tumour and adjacent normal tissues have begun to reveal structured microbial alterations associated with oral cavity squamous cell carcinoma. By coupling taxonomic indices with spatial and host-interface features, future studies may better understand whether specific microbial consortia function as passive biomarkers or active contributors to carcinogenic microenvironments [65].

## Conclusions and perspectives

Investigating the link between the native spatial architecture of the microbiota and its function requires sampling strategies that maintain microbial populations within their original topology. Such approaches enable simultaneous visualisation of microorganisms and host structures across scales. Conventional thin-section imaging, however, fails to fully capture the spatial complexity of microbial communities. Alternative low-perturbation tools, such as ridged plastic tongue scrapers, can recover biofilm fragments that retain spatial organisation, although compression during collection remains a limitation [6,66]. Recent advances include tissue-clearing techniques that maintain the spatial integrity of mucosal microbiota, host tissues, and the fragile mucus layer [67]. Despite this progress, the method has yet to be applied to oral biofilms or integrated into clinical patient sample workflows, which needs further validation.

*In vitro* models replicating the in-situ microenvironment have emerged to study spatial interactions within the oral microbiome. Although short-range factors such as direct adhesion and steep micron-scale gradients yield a powerful influence on microbial niches, longer-range factors likewise shape oral biofilm composition in complex ways. Interactions might occur between taxa separated by centimeters across the mouth through small molecules diffusing in air or transported via salivary flow [66]. Spatiotemporal tracking of microbial populations similarly offers further insights into how these interspecies interactions evolve and drive broader shifts in microbial architecture over time [68]. Moreover, organs-on-a-chip approach combined with microfluidics enables real-time characterisation of biofilm morphology, colonisation density, and spatial arrangement with *in vitro* models mimicking human oral conditions including salivary flow and different niches [69]. By reconstructing high-resolution three-dimensional or time-lapse maps, these approaches reveal how local niches shape microbial ecology at the microscale, ultimately influencing transitions from health- to disease-associated communities [70].

However, imaging-based findings have inherent limitations. Despite technical advances, microscopy remains fundamentally descriptive, signals are influenced by labelling strategies, photophysics, sampling/fixation artifacts, image processing, and downstream analytic pipelines, making results vulnerable to confounding and bias. Accordingly, targeted genetic or chemical perturbations, controlled *in vitro* assays, and *in vivo* or clinical studies are required to validate observations and establish causality.

The spatial and functional specificity of chemical interactions such as metabolites within the oral cavity underscores their potential as both diagnostic biomarkers and therapeutic targets. Advances in spatial metabolomics have revealed location-specific metabolites, highlighting the importance of micro-environmental factors in shaping microbial interactions. The future of oral health management lies in precision microbiome modulation, where spatially and functionally relevant metabolites serve as both early disease indicators and therapeutic targets, promoting a balanced oral microbiome. Integrating AI-driven analyses with advanced imaging and multi-omics approaches can create predictive models that simulate the effects of microbial interaction, metabolite production and host response on community stability and health outcomes, which can lead to more accurate predictions of disease progression and personalised therapeutics (Figure 4).

## Data Availability

Making data available does not apply to this review paper.
